# A high-affinity potassium transporter (MeHKT1) from cassava (*Manihot esculenta*) negatively regulates the response of transgenic *Arabidopsis* to salt stress

**DOI:** 10.1186/s12870-024-05084-7

**Published:** 2024-05-07

**Authors:** Minghua Luo, Jing Chu, Yu Wang, Jingyan Chang, Yang Zhou, Xingyu Jiang

**Affiliations:** 1 National Center for Technology Innovation of Saline-Alkali tolerant Rice, College of Coastal Agricultural Sciences, Guangdong Ocean University, Zhanjiang, 524088 China; 2 Key Laboratory for Quality Regulation of Tropical Horticultural Crops of Hainan Province, School of Life and Health Sciences, Hainan University, Haikou, 570228 China

**Keywords:** Cassava, High-affinity potassium transporter, Potassium starvation, Salt tolerance

## Abstract

**Background:**

High-affinity potassium transporters (HKTs) are crucial in facilitating potassium uptake by plants. Many types of HKTs confer salt tolerance to plants through regulating K^+^ and Na^+^ homeostasis under salinity stress. However, their specific functions in cassava (*Manihot esculenta*) remain unclear.

**Results:**

Herein, an *HKT* gene (*MeHKT1*) was cloned from cassava, and its expression is triggered by exposure to salt stress. The expression of a plasma membrane-bound protein functions as transporter to rescue a low potassium (K^+^) sensitivity of yeast mutant strain, but the complementation of MeHKT1 is inhibited by NaCl treatment. Under low K^+^ stress, transgenic *Arabidopsis* with *MeHKT1* exhibits improved growth due to increasing shoot K^+^ content. In contrast, transgenic *Arabidopsis* accumulates more Na^+^ under salt stress than wild-type (WT) plants. Nevertheless, the differences in K^+^ content between transgenic and WT plants are not significant. Additionally, *Arabidopsis* expressing *MeHKT1* displayed a stronger salt-sensitive phenotype.

**Conclusion:**

These results suggest that under low K^+^ condition, MeHKT1 functions as a potassium transporter. In contrast, MeHKT1 mainly transports Na^+^ into cells under salt stress condition and negatively regulates the response of transgenic *Arabidopsis* to salt stress. Our results provide a reference for further research on the function of *MeHKT1*, and provide a basis for further application of *MeHKT1* in cassava by molecular biological means.

**Supplementary Information:**

The online version contains supplementary material available at 10.1186/s12870-024-05084-7.

## Background

Potassium (K^+^) is the primary cation within plant cells, constituting approximately 2–10% of the dry weight of plants. Therefore, it is a crucial nutrient sustaining plant growth and development [1]. K^+^ is pivotal in many physiological and biochemical processes, such as osmotic regulation, enzyme activation, charge neutralization, and maintenance of membrane potential in plant cells [2]. Moreover, K^+^ regulates photosynthesis, starch synthesis, and subsequent transport and metabolism of carbohydrates. This regulation enhances grain quality and increases crop yields [3, 4]. In addition, ensuring a sufficient supply of K^+^ can bolster crop resilience against several biotic and abiotic stresses, including drought and salt [4]. However, most of the world’s arable land faces K^+^ deficiency, severely limiting the sustainable development of agricultural production [5]. Sodium (Na^+^) is a non-essential element with chemical and physical properties similar to those of K^+^. Na^+^ competes with K^+^ for uptake through the plasma membrane of the plant root system, leading to cell depolarization. This process elevates K^+^ leakage, decreases the K^+^/Na^+^ ratio, and ultimately causes plant salt injury [6]. Moreover, soil salinization is one of the major factors restricting land use, leading to remarkable reductions in global crop yields [7–9]. Upon salt stress exposure, the accumulation of excessive Na^+^ in plants leads to physiological disruptions. Therefore, it is crucial to prioritize the maintenance of a low level of Na^+^ or prevent Na^+^ from entering the cytosol of plant cells to protect them from damage in saline soils. Plants have developed multiple strategies to cope with salt stress in response to excess Na^+^ [10, 11]. These strategies include transporting Na^+^ from the upper parts of the plant to the roots and returning Na^+^ to the soil solution. One such mechanism involves the action of salt overly sensitive 1 (SOS1) antiporter [12]. Moreover, plants sequester Na^+^ into vacuoles via sodium proton antiporter (NHX) to reduce sodium accumulation in the cytoplasm [13]. Another way is to retrieve Na^+^ from the xylem stream via a high-affinity potassium transporter (HKT) to reduce Na^+^ accumulation in shoots [14].

The HKT proteins consist of four MPM repeats (M1A-PA-M2A-M1D-PD-M2D). “M” denotes the transmembrane segment, while “P” signifies the pore-loop domain. These repeats are assembled to form permeation pathways and function in a similar way to K^+^ channels. In the last three of the four pore-loop domains, specifically from PB to PD, all HKT proteins contain the GYG motif. This motif is strongly conserved in the selective filters of K^+^ transporters. However, there are variations in the first position of the conserved PA motif. Through phylogenetic and functional assessments, the HKT family in plants can be classified into two distinct subfamilies: subfamily I (SGGG-type) and subfamily II (GGGG-type). The former, which mainly transports Na^+^ selectively, is widely distributed in monocots and dicots. It notably features a serine residue in the first p-loop rather than a typical glycine residue. On the other hand, the second subfamily, capable of permitting the passage of either K^+^ or Na^+^ and can even function as a Na^+^-K^+^ symporter, has only been identified in monocots. In this subfamily, a glycine residue is present in the first p-loop [15–18]. Some exceptions to this rule were reported, suggesting these amino acids could be involved transporting specific ions mediated by HKT in plants [18, 19].

Many HKT1-type transporters were identified and thoroughly studied in various plant species. AtHKT1;1, only one member of HKT subfamily I in *Arabidopsis thaliana*, is primarily found in the plasma membrane of vascular parenchyma cells surrounding the xylem [20, 21]. It specifically transports Na^+^ when it exhibits heterologous expression in *Xenopus laevis* oocytes [22] and functions in retrieving Na^+^ from the xylem, thereby lowering Na^+^ accumulation in shoots. The importance of AtHKT1;1 in salt tolerance of *Arabidopsis* has been reported [14, 23], and it also contributes to the natural selection of salt-tolerant *Arabidopsis* accession [24]. OsHKT1;5, an orthologs of AtHKT1;1, is first identified as a quantitative trait locus of SKC1 in *Oryza sativa* [25] that encodes a HKT-type transporter capable of unloading Na^+^ from the root xylem [26]. The *oshkt1;5* mutant accumulates more Na^+^ in the shoot than wild-type (WT) plants. This observation suggests that OsHKT1;5 facilitates the elimination of Na^+^ in the vasculature under saline conditions. This function helps safeguard the leaves and reproductive system of rice from Na^+^ toxicity [27]. Notably, the functions of OsHKT1;1 and OsHKT1;4 are similar to that of OsHKT1;5 [28, 29]. Furthermore, TmHKT1;5-A and TaHKT1;5-D are the target loci of Nax2 and Kna1 from *Triticum monococcum* and *Triticum aestivum*, respectively. These loci are pivotal in excluding Na^+^ and enhancing salt tolerance [30, 31]. In addition, SlHKT1;2 and RtHKT1 regulate Na^+^/K^+^ homeostasis and enhance salt tolerance of *Solanum lycopersicum* and *Reaumuria trigyna* [32, 33]. However, the barley (*Hordeum vulgare*) HKT transporter, HvHTK1;5, serves a distinct physiological function. When HvHTK1;5 is subjected to RNA interference (RNAi) in barley, it remarkably reduces Na^+^ transport from roots to shoots. Consequently, this increases the K^+^/Na^+^ ratio compared to WT plants under salt stress, exhibiting a more pronounced salt-tolerant phenotype [34].

Cassava (*Manihot esculenta* Crantz) mainly grows in tropical and sub-tropical regions and ranks as the fourth most crucial staple crop in Africa [35]. Cassava holds immense economic value in alleviating poverty in developing countries, particularly in regions with poor soil quality and arid conditions. This is owing to its remarkable ability to adapt to adverse environments, surpassing the resilience of many other crops. It was demonstrated that low K^+^ stress has a remarkable impact on the yield and quality of cassava [36]. However, the precise mechanism underlying K^+^ nutrition in cassava remains unclear. In order to study the response mechanisms of cassava to low potassium stress and provide reference for cassava to use potassium nutrition efficiently. Herein, an HKT-type gene (*MeHKT1*) is cloned from cassava, and its function was elucidated via heterologous expression in yeast and *Arabidopsis*. The findings suggest that the MeHKT1 transporter primarily facilitates K^+^ uptake when exposed to low K^+^ conditions. However, MeHKT1 primarily functions in the transport of Na^+^ and has a negative regulatory function in plant salt tolerance under salt stress conditions.

## Results

### Cloning and bioinformatics analysis of MeHKT1

**Fig. 1:**
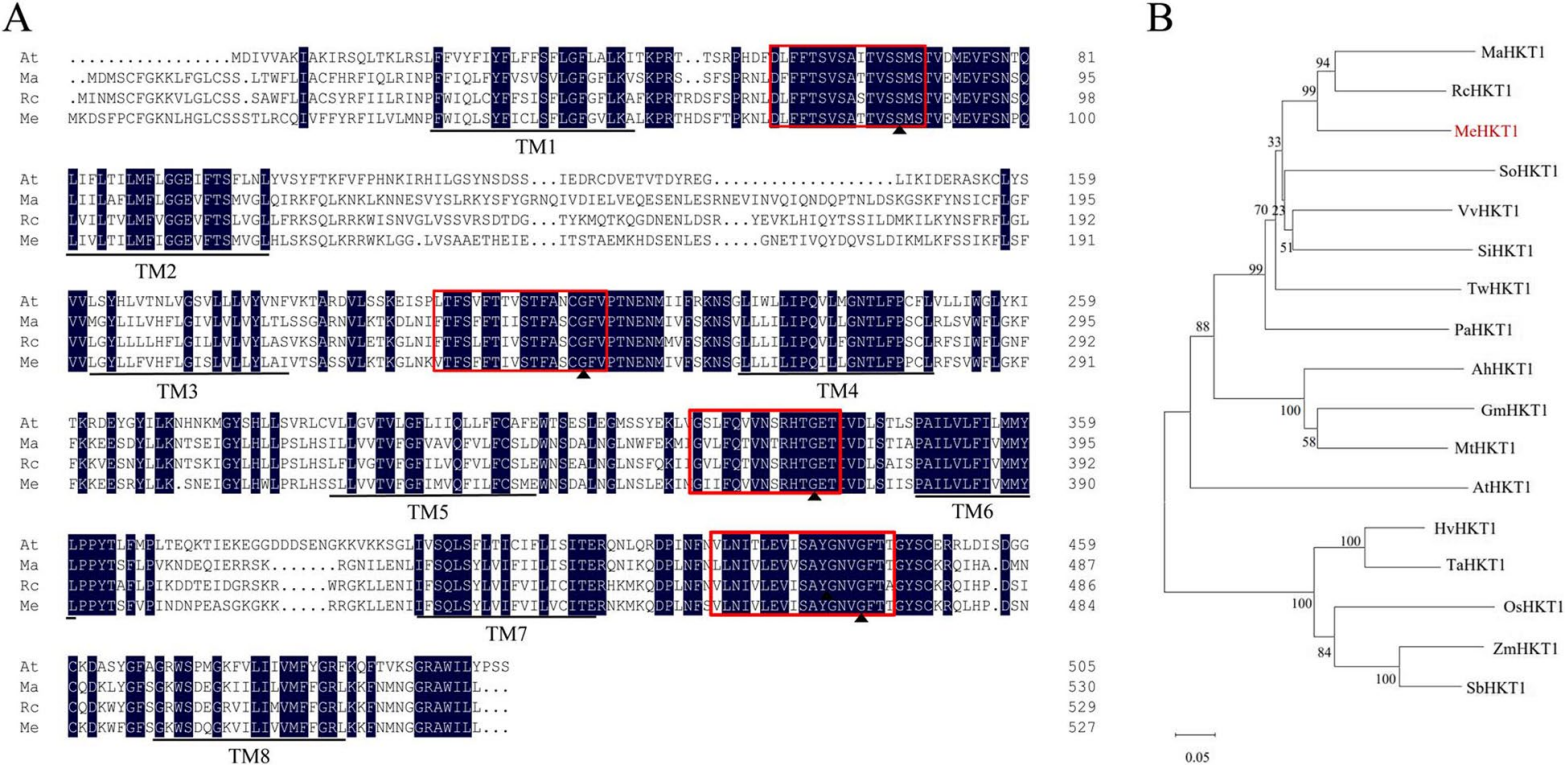
Alignment and phylogeny of HKT subfamily members. **A** Multiple alignments of the deduced amino acid sequences of the HKT proteins from *Arabidopsis thaliana* (At, KAG7615472.1), *Mercurialis annua* (Ma, XP_050227445.1), *Ricinus communis* (Rc, XP_015577806.1), and *Manihot esculenta* (Me, XP_021620110.1). Four highly conserved pore domains are represented by red boxes, conserved serine, and glycine residues by solid triangles, and the 8 putative transmembrane domains by lines. **B** Phylogenetic analysis of the HKT subfamily transporters. The protein sequences are as follows: *Mercurialis annua* (MaHKT1, XP_050227445.1), *Ricinus communis* (RcHKT1, XP_015577806.1), *Manihot esculenta* (MeHKT1, XP_021620110.1), *Syzygium oleosum* (SoHKT1, XP_030473997.1), *Vitis vinifera* (VvHKT1, RVW85979.1), *Sesamum indicum* (SiHKT1, XP_011077901.1), *Tripterygium wilfordii* (TwHKT1, XP_038700095.1), *Potentilla anserina* (PaHKT1, XP_050375358.1), *Arachis hypogaea* (AhHKT1, XP_025700773.1), *Glycine max* (GmHKT1, XP_014620373.3), *Medicago truncatula* (MtHKT1, KEH27275.1), *Arabidopsis thaliana* (AtHKT1, KAG7615472.1), *Hordeum vulgare* (HvHKT1, KAE8777465.1), *Triticum aestivum* (TaHKT1, ABG33945.1), *Oryza sativa* (OsHKT1, AFY08296.1), *Zea mays* (ZmHKT1, PWZ31832.1), and *Sorghum bicolor* (SbHKT1, 002457736.2). The scale bar illustrates a length representing 0.05 of the value

*MeHKT1* was cloned from cassava with a full-length CDS of 1584 bp, encoding 527 amino acid residues with a predicted molecular weight (MW) of 59.54 kDa and a predicted theoretical isoelectric point (pI) of 9.28. The sequence of MeHKT1 underwent alignment with HKT1 proteins from other plants, revealing the existence of eight putative transmembrane domains and four p-loop domains that were highly conserved. Notably, MeHKT1 proteins exhibited the conservation of serine (S) residues in the first p-loop domain and glycine (G) residues in the last three p-loop domains. This conservation pattern indicates cassava MeHKT1 belongs to the HKT subfamily I (SGGG type). In comparison, MeHKT1 shares a substantial sequence identity of 73% with RcHKT1 (*Ricinus communis*), 69% with MaHKT1 (*Mercurialis annua*), and 51% with AtHKT1 (*Arabidopsis thaliana*) (Fig. 1A; Figure S1). The outcomes of the phylogenetic tree analysis revealed a distinct grouping pattern. HvHKT1 (*Hordeum vulgare*) from barley, TaHKT1 (*Triticum aestivum*) from wheat, OsHKT1 (*Oryza sativa*) from rice, ZmHKT1 (*Zea mays*) from maize, and SbHKT1 (*Sorghum bicolor*) from sorghum, all of which are monocotyledons, formed a cohesive cluster. In contrast, the other dicotyledons clustered in another group. Moreover, MeHKT1 from cassava was closely related to RcHKT1 from *Ricinus communis* and MaHKT1 from *Mercurialis annua* (Fig. 1B).

### Expression analysis of MeHKT1 from cassava

**Fig. 2:**
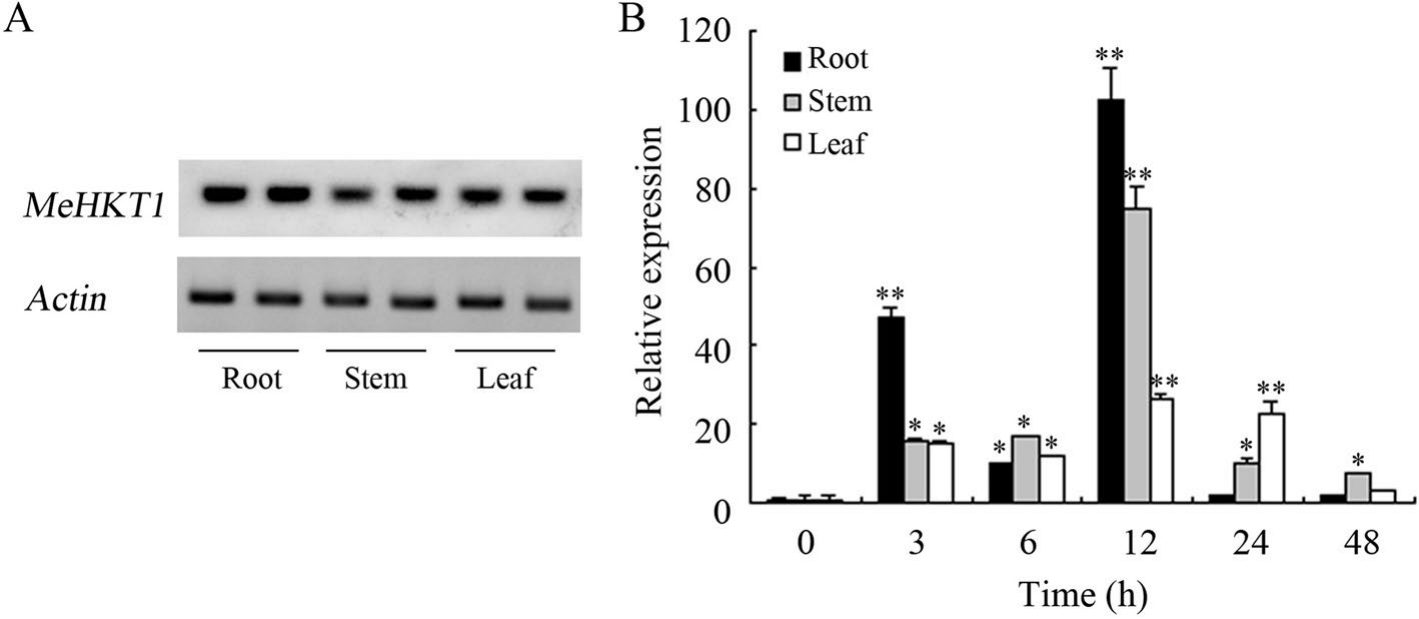
The expression patterns of the *MeHKT1* gene in cassava. **A** RT-PCR analysis of *MeHKT1* gene in various tissues of cassava. **B** *MeHKT1* gene expression analysis in various cassava tissues under 150mM NaCl treatment for 48 h. *Actin* was used as an internal control. Data are expressed as the mean ± SD of three replicates. Asterisks (* and **) indicate significant difference by one-way ANOVA (*P* < 0.05 and *P* < 0.01)

The tissue expression analysis of *MeHKT1* revealed its presence in roots, stems and leaves, all exhibiting similar expression levels without any remarkable differences (Fig. 2A). To delve deeper into understanding the response of *MeHKT1* under stress conditions, cassava plants were exposed to either low potassium (K^+^) or high-salinity stress. Following that, *MeHKT1* transcript levels were examined utilizing a qRT-PCR. The outcomes indicated that the expressions of *MeHKT1* in all examined tissues remained unaffected by K^+^ starvation (data not shown). However, when subjected to salt stress, the transcript levels in roots, stems and leaves exhibited upregulation. This upregulation reached its peak value after 12 h of salt treatment, showing an increase of 102.5-fold, 74.8-fold, and 26.3-fold, respectively, compared to the levels at 0 h. Subsequently, the expression levels declined but remained higher than those observed at the initial 0 h time point (Fig. 2B).

### Subcellular localization of MeHKT1 protein

**Fig. 3:**
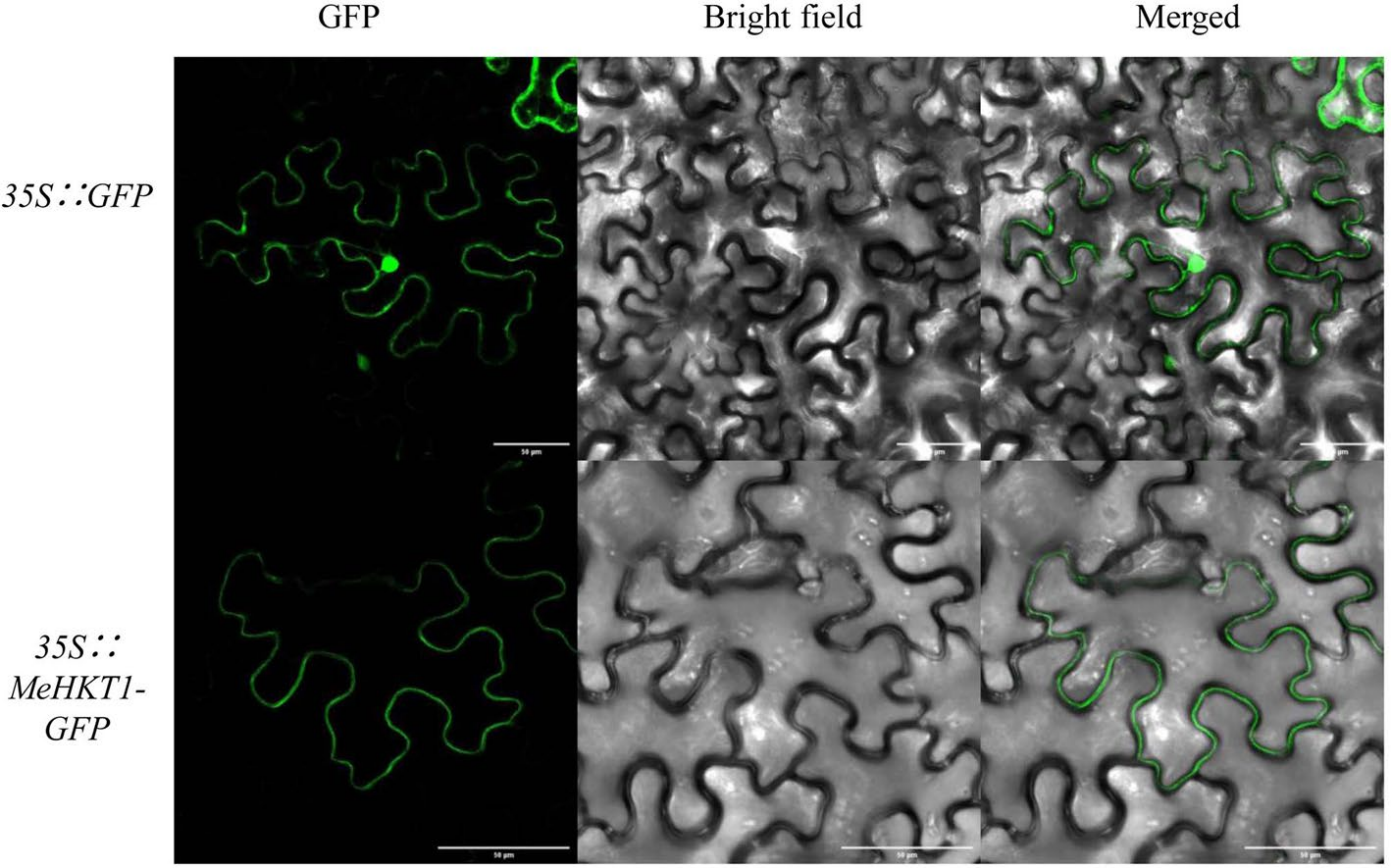
Subcellular localization of MeHKT1 protein in *Nicotiana benthamiana* leaf epidermis cells. Tobacco leaves were transformed with the vector pCAMBIA1300 containing 35 S::MeHKT1-GFP or 35 S::GFP. After incubation for 3 days, GFP signals in the tobacco leaf epidermis cells expressing 35 S::GFP or 35 S::MeHKT1-GFP were recorded using confocal microscopy. All panels in this figure have 50 μm scale bars

The presence of 8 putative transmembrane domains in the MeHKT1 protein (Supplementary Figure S1) confirmed its classification as a membrane-bound protein. To investigate the subcellular localization of MeHKT1, the transient expression of MeHKT1-GFP fusion protein was observed after injection into tobacco leaves for 3 days. The transient expression of GFP harbored by the construct 35 S::GFP served as a control. The fluorescent signals of GFP were observed both at the cell membrane and intracellularly in tobacco epidermal cells. In contrast, the fluorescent signals from MeHKT1-GFP fusion protein were detected on the cell membrane (Fig. 3). These findings strongly suggested that MeHKT1 was localized on the cell membrane.

### Functional analysis of MeHKT1 in yeast

**Fig. 4:**
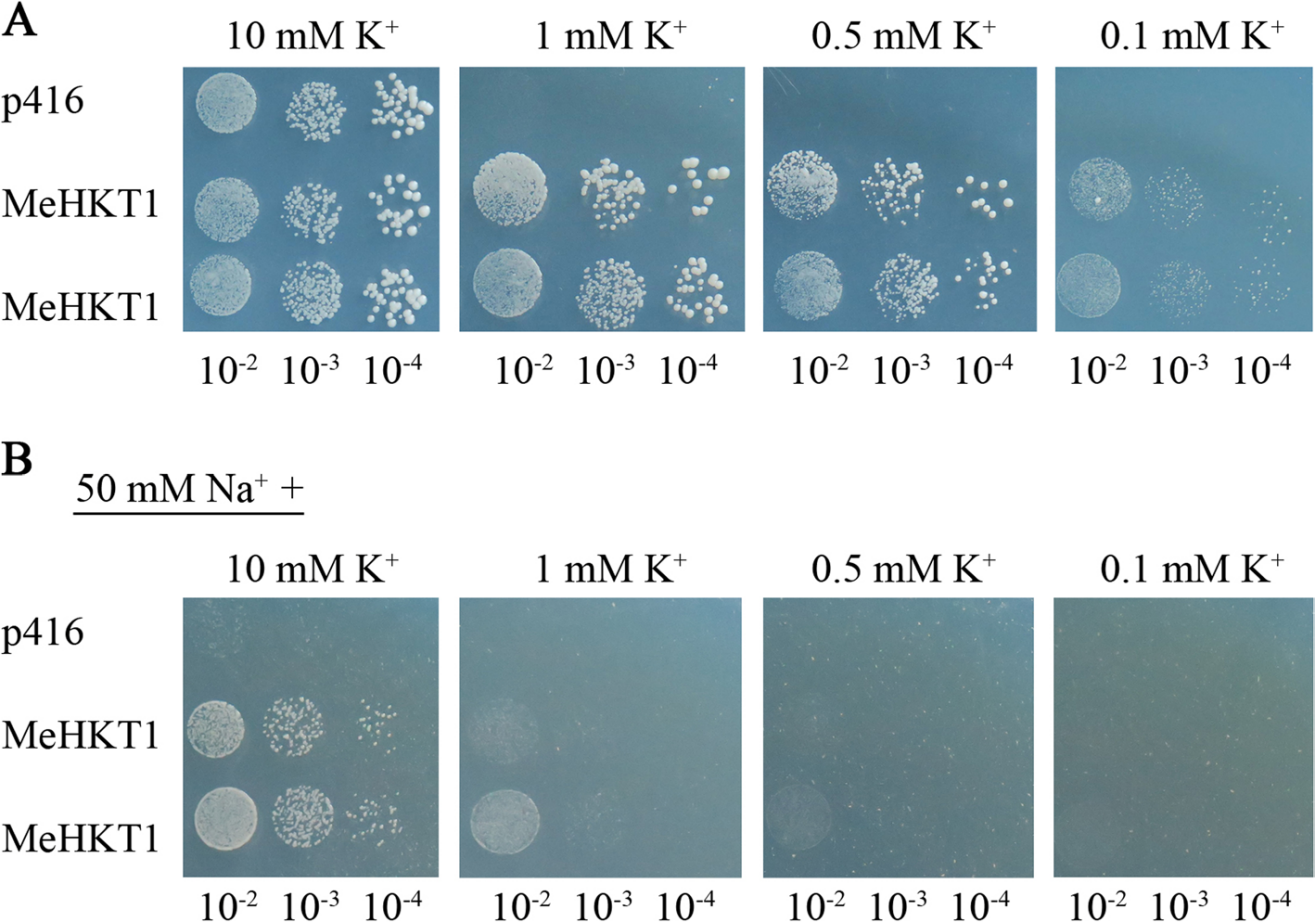
MeHKT1 expression in the yeast mutant strain CY162. Functional analysis of MeHKT1 in the mutant yeast strain CY162, yeast cells with empty vector p416 as control. **A** Transgenic yeast cells were spotted on AP plates containing different concentrations of KCl as indicated and allowed to grow at 28 °C for 3 d. **B** Transgenic yeast cells were spotted on AP plates containing 50 mM NaCl and different concentrations of KCl as indicated and allowed to grow at 28 °C for 3 d

Yeast strain CY162(*trk1Δ*, *trk2Δ*) represents a K^+^ uptake-deficient yeast mutant strain. This strain exhibits inhibited growth when the K^+^ concentration falls below 5 mM, making it a valuable tool for verifying the functionality of K^+^ uptake in a heterologous system. There was no remarkable disparity in the growth of CY162 transformed with *MeHKT1* and empty vector when the K^+^ concentration was 10 mM. However, as the K^+^ concentration decreased, the yeast strain growth transformed with the empty vector control was significantly suppressed. At K^+^ concentrations below 1 mM, the empty vector transformants exhibited significant growth impairment, while the *MeHKT1* yeast transformants maintained robust growth. Moreover, even when the K^+^ concentration was reduced to 0.1 mM, the *MeHKT1* yeast transformants successfully restored the growth of the yeast strain CY162 (Fig. 4A). Moreover, the growth phenotype of transgenic yeast cells exposed to 0.5 mM KCl was similar to that under 10 mM KCl. These findings indicated that in low K^+^ environments, MeHKT1 served as a K^+^ uptake transporter and improved the yeast mutant strain sensitivity at high-affinity conditions.

To determine the effect of high external concentrations of Na^+^ on the K^+^ transport activity of MeHKT1, *MeHKT1* yeast transformants were spotted onto AP solid medium containing 50 mM NaCl at different K^+^ concentrations. The empty vector transformants were employed as the control. At a K^+^ concentration of 10 mM, the presence of 50 mM NaCl had a remarkable inhibitory effect on the growth of empty vector transformants. In contrast, the growth of *MeHKT1* transformants was unaffected by 50 mM NaCl at this K^+^ concentration, displaying no difference compared with the control. However, when the K^+^ concentration dropped below 1 mM, the growth of *MeHKT1* transformants was notably inhibited by 50 mM NaCl, and this inhibition was significantly different from the control (Fig. 4B). These findings strongly indicated that the K^+^ transporter activity of MeHKT1 is significantly suppressed by the presence of 50 mM NaCl under conditions of low K^+^.

### MeHKT1 overexpression positively regulated the response of transgenic Arabidopsis to low K^+^ stress

**Fig. 5:**
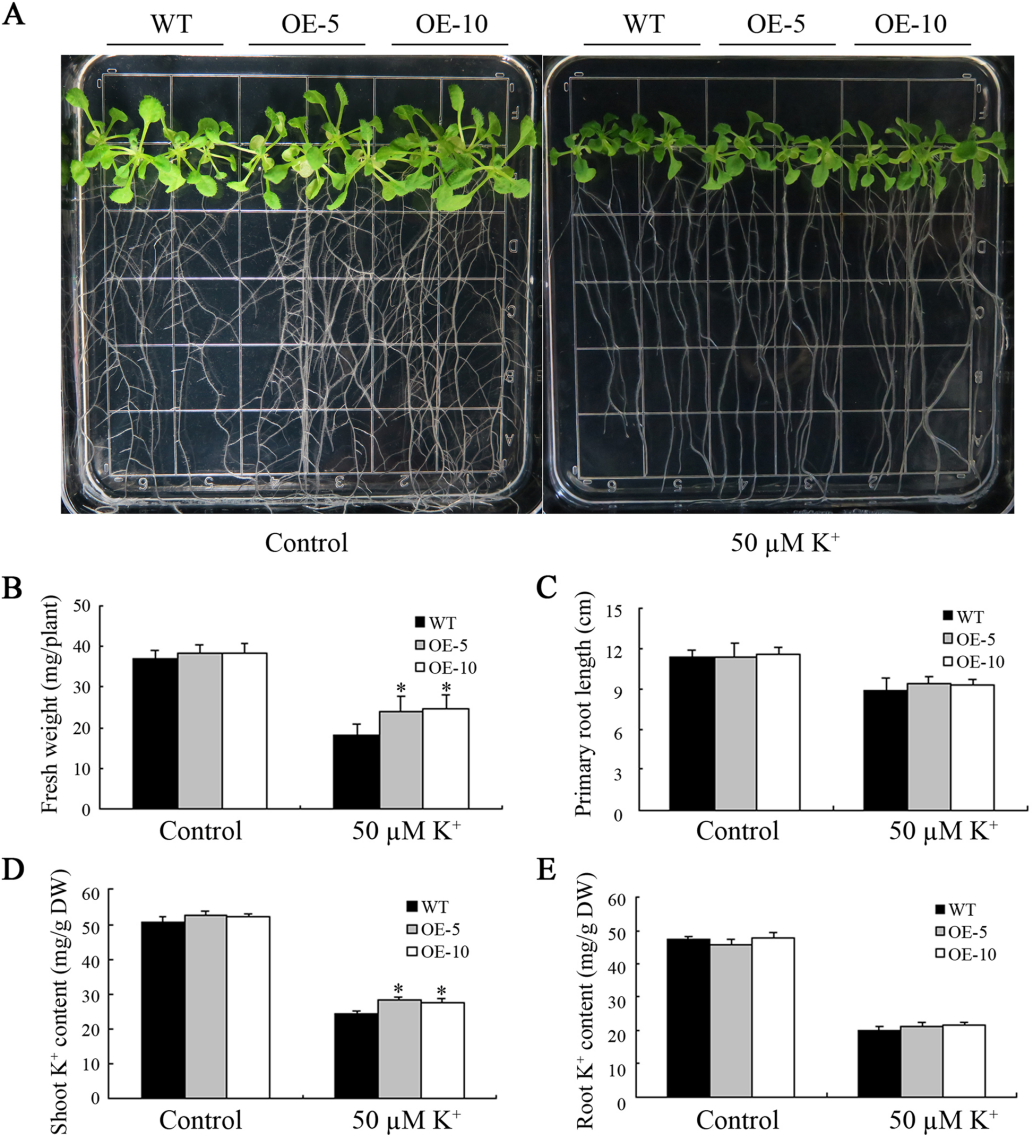
Functional analysis of *MeHKT1* in transgenic *Arabidopsis thaliana* under low K^+^ stress. The four-day-old wild-type (WT) and *MeHKT1* overexpressing (OE) seedlings were transferred to 1/2 MS medium with or without 50 µM KCl and their phenotypes (**A**) observed on the 14th day after transferring. Seedling fresh weight (**B**), primary root length (**C**), and K^+^ contents in shoots (**D**) and roots (**E**) of WT and OE plants are measured after low K^+^ stress for 14 d. Values are represented as means ± SD of four replicates. Asterisks (*) represent significant differences based on one-way ANOVA (*P* < 0.05)

To assess the involvement of MeHKT1 in a K^+^ deficient environment, both wild-type (WT) and *MeHKT1* transgenic (OE) *Arabidopsis* were initially germinated on 1/2 MS medium for a period of 4 days. Subsequently, they were transplanted onto two different media: one with low potassium (LK) conditions containing 50 µM K^+^ and the other serving as a control, which consisted of 1/2 MS medium. The findings indicated no significant disparity between the growth characteristics of WT and OE plants on 1/2 MS medium. However, OE plants exhibited significantly improved growth compared to WT plants on the LK medium (Fig. 5A). Further analysis of relevant parameters revealed that, under controlled conditions, no significant differences in terms of fresh weight or primary root length were observed between WT and OE plants. Nevertheless, after exposure to LK stress conditions, the seedling fresh weight of OE plants exhibited a substantial increase relative to WT (Fig. 5B). Additionally, the primary root length of OE plants was marginally larger in comparison to WT (Fig. 5C). During control conditions, there were no substantial variations in K^+^ content between the shoots and roots of WT and OE. Furthermore, due to LK stress, the K^+^ content in the shoot of OE plants was notably elevated compared to WT. However, the disparity in K^+^ content between the roots of WT and OE plants did not reach statistical significance (Fig. 5D and E). These outcomes strongly indicated that OE plants enhanced K^+^ uptake under K^+^-deficient conditions.

### Functional characterization of MeHKT1 in transgenic Arabidopsis under salt stress

**Fig. 6:**
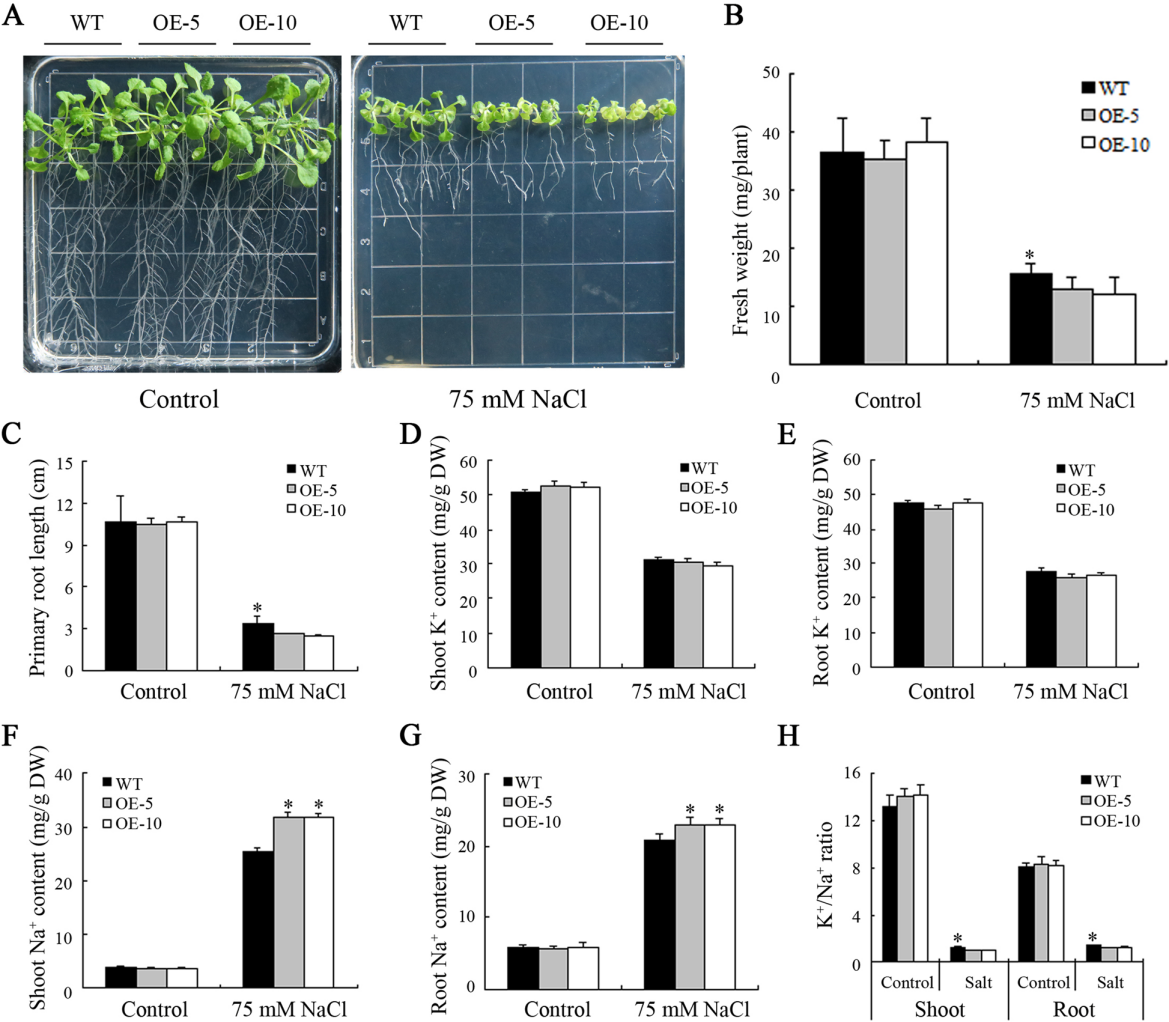
Functional analysis of *MeHKT1* in transgenic *Arabidopsis thaliana* under salt stress. The four-day-old wild-type (WT) and *MeHKT1* overexpressing (OE) seedlings were transferred to 1/2 MS medium with or without 75 mM NaCl and their phenotypes (**A**) observed on the 14th day after transferring. Seedling fresh weight (**B**), primary root length (**C**), and (**D**, **E**) potassium (K^+^) and (**F**, **G**) sodium (Na^+^) contents of WT and OE plants are determined in response to 75 mM NaCl treatment for 14 d, and K^+^/Na^+^ ratios (**H**) were calculated. Values are represented as means ± SD of four replicates. Asterisks (*) represent considerable variations by one-way ANOVA (*P* < 0.05)

In order to assess the performance of *MeHKT1* transgenic *Arabidopsis* under salt stress, the seeds of WT and OE plants were planted into 1/2 MS medium. These seedlings were subsequently grown in a plant light incubator for a period of 4 d. After this initial growth phase, they were transplanted into two different growth environments: one with 1/2 MS medium having 75 mM NaCl and the other using 1/2 MS medium as a control. The plants continued to grow in salt-stress conditions in the plant light incubator for 14 d to compare salt tolerance phenotypes between WT and OE plants. There were no remarkable variations in the growth phenotypes of WT and OE plants under a controlled environment. Nevertheless, under salt stress, the growth of OE plants was remarkably weaker in comparison to WT (Fig. 6A). Simultaneously, it was noted that the fresh weight of OE plants exposed to salt stress was remarkably lower relative to WT plants (Fig. 6B). Furthermore, the primary root length of OE plants was remarkably shorter than that of WT plants under the same salt stress conditions (Fig. 6C). Additional analysis of K^+^ and Na^+^ contents revealed no remarkable disparity between WT and OE plants in terms of K^+^ and Na^+^ contents in shoots and roots under control conditions. However, under salt-stress conditions, OE plants exhibited notably elevated Na^+^ contents in shoots and roots than those of WT plants. As a result, the K^+^/Na^+^ ratios in the shoots and roots of OE plants were much lower than those of WT plants (Fig. 6D-H). The outcomes indicated that the salt tolerance of WT *Arabidopsis* was notably better than *MeHKT1* transgenic *Arabidopsis*.

### MeHKT1 overexpression negatively regulated salt tolerance of transgenic Arabidopsis plants grown in Soil

**Fig. 7:**
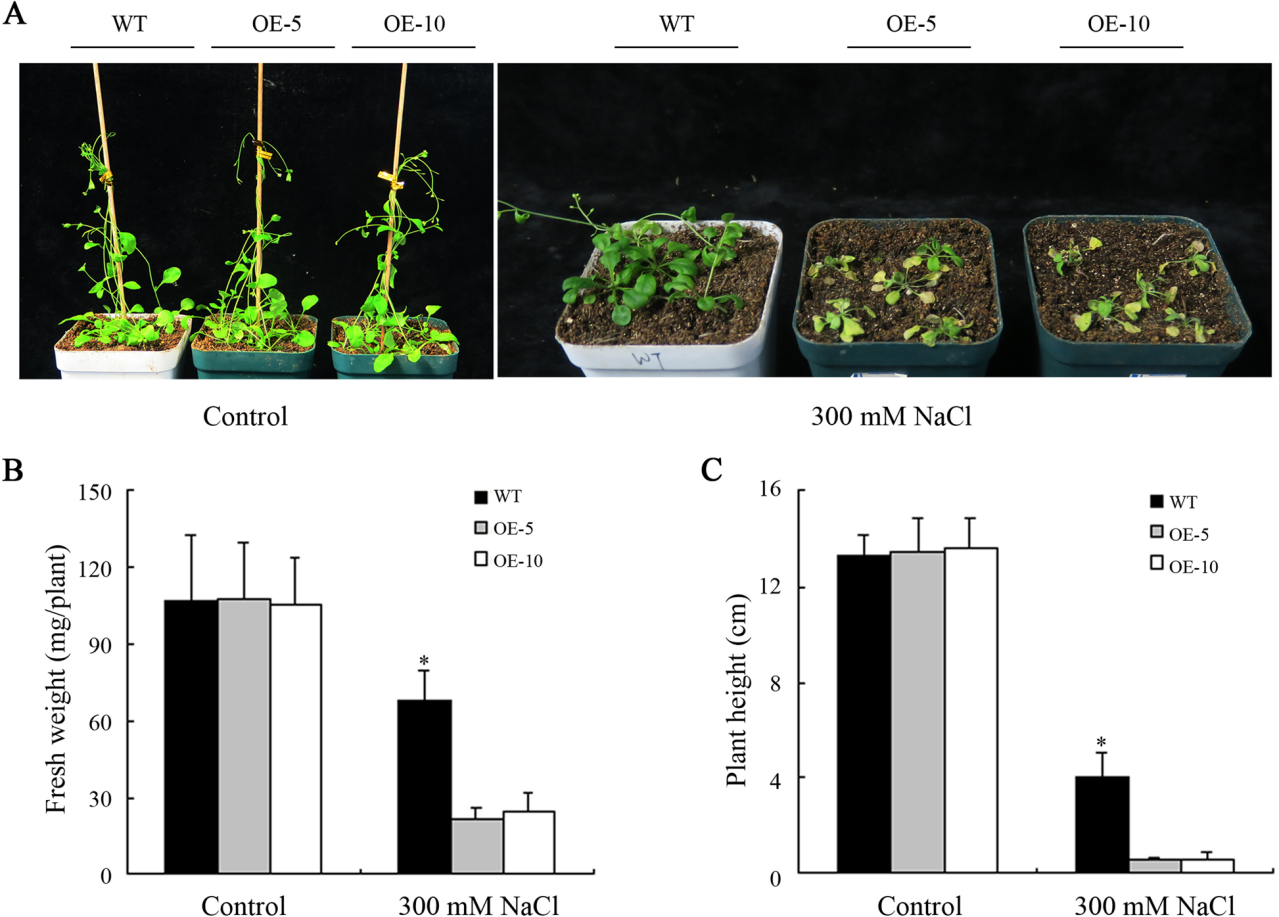
Functional analysis of *MeHKT1* in transgenic *Arabidopsis thaliana* grown in the soil under salt stress. The 13-d-old wild-type (WT) and *MeHKT1* overexpressing (OE) seedlings were transferred in soils with or without 300 mM NaCl and allowed to grow for 21 d, and then their phenotypes (**A**) observed, shoot fresh weight (**B**) and plant height (**C**) were determined. Values are represented as means ± SD of five replicates. Asterisks (*) represent significant differences by one-way ANOVA (*P* < 0.05)

Salt tolerance assays of WT and OE plants in soil were performed to further examine the function of MeHKT1 in salt response. Under control conditions, the growth phenotypes of WT and OE plants were similar. However, when subjected to watering with 300 mM NaCl, the growth of OE plants was noticeably weaker compared to that of WT plants. Moreover, the growth of OE plants was substantially inhibited, exhibiting signs of wilting and yellowing, whereas the WT plants retained their dark green appearance and continued to grow well (Fig. 7A). Additionally, under the salt treatment conditions, the height of OE plants watered with 300 mM NaCl was considerably lower than that of WT. Moreover, the seedling fresh weight of OE plants was considerably lower in comparison to WT (Fig. 7B and C).

**Fig. 8:**
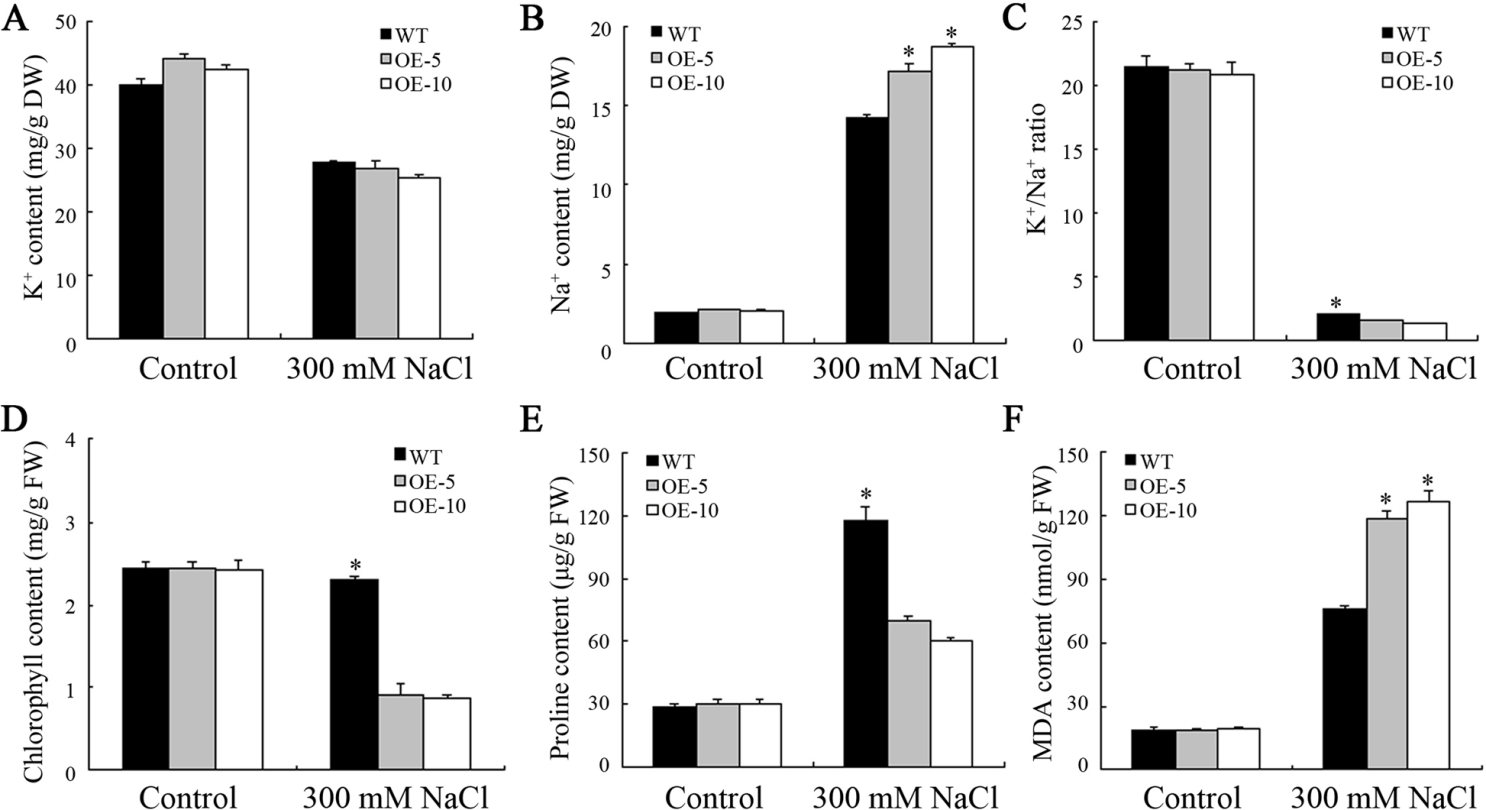
Physiological parameters of *MeHKT1* transgenic *Arabidopsis thaliana* grown in the soil under salt stress. The 13-d-old wild-type (WT) and *MeHKT1* overexpressing (OE) seedlings were transferred in soils with or without 300 mM NaCl and allowed to grow for 21 d, and then their K^+^ (**A**) and Na^+^ (**B**) contents, K^+^/Na^+^ ratio (**C**), chlorophyll (**D**), proline (**E**), and malondialdehyde (MDA) (**F**) contents were determined. Values are represented as means ± SD of three replicates. Asterisks (*) denote significant differences by one-way ANOVA (*P* < 0.05)

Subsequently, the K^+^ and Na^+^ concentrations of WT and OE plants were analyzed. Under normal conditions, there were no substantial differences in the K^+^ and Na^+^ contents between WT and OE plants. However, the Na^+^ content of OE plants watered with 300 mM NaCl was considerably elevated than that of WT. Moreover, the K^+^/Na^+^ ratio of OE plants was also considerably less than that of WT (Fig. 8A-C). Furthermore, the chlorophyll, MDA, and proline contents of WT and OE plants were determined. Under salt stress, the OE plants showed remarkably lower chlorophyll and proline contents than those of WT. However, the MDA content was considerably elevated in comparison to WT. Under normal conditions, these parameters were essentially identical in both WT and OE plants (Fig. 8D-F).

The findings mentioned above align with the results observed in WT and OE plants on medium, further confirming that *MeHKT1* transgenic plants exhibit significantly greater salt sensitivity than WT plants.

## Discussion

Previous studies have reported different expression patterns of *HKT1*s in various plants. In the case of rice, *OsHKT1;1* demonstrates a predominant expression in the phloem of leaf blades, with its transcripts being induced in shoots while remaining unaltered in roots [18, 28]. On the other hand, *OsHKT1;5* exhibits a predominant expression profile in the parenchyma cells that envelop the xylem vessels within roots. Moreover, its transcripts exhibit an upregulation in roots and basal stems in response to salt stress conditions [26, 27]. Moreover, *OsHKT1;4* transcripts are abundant in leaf sheaths at all stages of growth. Unexpectedly, during the reproductive stage, their transcripts accumulate in the stems [29]. In wheat, the *OsHKT1;5-like* gene *TmHKT1;5-A* exhibits constitutive expression in roots but remains uninduced by NaCl [37]. In barley, *HvHKT1;5* exhibits predominant expression in roots, and the level of its transcripts is induced because of the salt stress [34]. Conversely, in *Arabidopsis*, *AtHKT1;1* is primarily expressed in shoots, and its expression can be notably induced by mild salt stress, as reported in previous studies [21, 38, 39]. Interestingly, in halophytic *Arabidopsis* relatives, *TsHKT1;2* (*Thellungiella salsuginea HKT1;2*) and *EpHKT1;2* (*Eutrema parvula HKT1;2*) exhibit remarkable upregulation in response to salt stress. Moreover, *TsHKT1*;2 and *EpHKT1*;2 serve as potassium (K^+^) absorbers in these species [40, 41]. In the case of *SvHKT1;1* from *Sporobolus virginicus*, its expression is triggered by extreme salt stress (500 mM NaCl) rather than by mild or moderate salt stress (< 300 mM NaCl). Additionally, the abundance of its transcripts is higher in shoots than in roots [42]. *RtHKT1* from *Reaumuria trigyna* primarily exhibits expression in both roots and leaves. Interestingly, its transcript levels experience a rapid escalation in response to either salt stress or low K^+^ conditions, with different tissue-specific expression patterns pre- and post-stress exposure [33]. However, cassava *MeHKT1* demonstrates a distinct expression profile compared to other *HKT1*s. Under normal conditions, it maintains similar expression level in roots, stems, and leaves. Notably, while a low K^+^ concentration dose not elicit such a response, only exposure to salt stress induces its transcript levels. Furthermore, the highest levels of its transcripts are observed in roots (Fig. 2, data not shown). Therefore, the distinct expression patterns noted in *HKT1*s across various plants have their own expression characterization, suggesting that they may possess different functions.

Studies have demonstrated that plant HKT1 exhibits transport properties correlated to the concentrations of Na^+^ and K^+^ in the external environment. For instance, when wheat *TaHKT1* exhibits expression in yeast or *Xenopus oocytes*, it acts as a Na^+^-K^+^ cotransporter when external concentrations of Na^+^ and K^+^ are in equilibrium. However, when the external Na^+^ concentration is excessively elevated, *TaHKT1* functions as a Na^+^-selective uniporter instead. A competitive binding model has been proposed to elucidate the dual mode of transport. Within the protein sequence of HKT1, there are high-affinity binding sites for K^+^ and Na^+^, and these ions engage in competitive binding with each other. The interplay and competition between K^+^ and Na^+^ at the K^+^ coupling site are pivotal in determining the specific ionic transport function of the transporter, dictating whether it functions as a K^+^-Na^+^ cotransporter or as a Na^+^ selective uniporter [43, 44]. It has been observed that RtHKT1 (*Reaumuria trigyna* HKT1) in transgenic *Arabidopsis* exhibits three distinct modes of transport properties, and these modes rely on the external Na^+^ or K^+^. Specifically, when external Na^+^ levels are high, RtHKT1 preferentially absorbs K^+^ to Na^+^. Under conditions of lesser external K^+^, RtHKT1 acts as a Na^+^-K^+^ cotransporter. Conversely, when external K^+^ concentrations are high, RtHKT1 operates as a Na^+^-selective uniporter. An intriguing observation is that the Na^+^ and K^+^ transport properties of RtHKT1 in transgenic yeast differ from those in transgenic *Arabidopsis* when subjected to higher levels of external Na^+^ or lower levels of external K^+^ conditions. However, their Na^+^/K^+^ transport properties align when exposed to high external K^+^ conditions. RtHKT1 acts as a Na^+^-K^+^ cotransporter in transgenic yeast cells under high external Na^+^ conditions. However, under lower external K^+^ conditions, it serves as a K^+^-selective transporter [33]. When exposed to high external Na^+^ concentrations or deficient K^+^, SbHKT1;4 predominantly operates as a Na^+^ transporter inside the *Arabidopsis* expression system. However, when external K^+^ levels are ample, it acts as a K^+^ transporter [45]. In contrast, AtHKT1;1 exhibits Na^+^ transport exclusively when external Na^+^ concentrations are elevated [20], and it remains non-conducting under low external K^+^ conditions [46]. *A. thaliana athkt1;1* mutant plants accumulated less Na^+^ in the phloem sap than wild type (WT) plants under salt stress, resulting in excessive accumulation of Na^+^ in aerial organs and less accumulation in roots, indicating that AtHKT1;1 could transport excess Na^+^ into roots through phloem under salt stress [47]. When *TmHKT1;5-A* and *TaHKT1;5-D* are introduced into *Xenopus laevis* oocytes, they act as dual-affinity transporters for Na^+^, capable of both high and low-affinity transport. However, the high-affinity transport function becomes inactive when the external K^+^ concentration surpasses that of external Na^+^. Additionally, the low-affinity transport function is also impeded by the presence of an external potassium ions supply (K^+^) [48]. In this study, it is evident that *MeHKT1* transgenic *Arabidopsis* accumulates higher levels of K^+^ in its shoots in comparison to the WT when subjected to low K^+^ conditions (Figs. 6 and 8). This suggests that MeHKT1 may be able to take up K^+^ under low K^+^ deficiency and functions as a K^+^-transporter. These outcomes align with results from previous studies on OsHKT2;1 from rice and HvHKT2;1 from barley [18, 49], but they diverge from previously reported observations regarding AtHKT1;1 from *Arabidopsis* and SbHKT1;4 from *S. bicolor* [45, 46]. In conditions of high external Na^+^, the transgenic *Arabidopsis* accumulates more Na^+^, whereas K^+^ content is not significantly different from that of WT. This observation suggests that MeHKT1 prefers Na^+^ transport to K^+^ and functions as a Na^+^-transporter under salt stress. However, this observation is inconsistent with the behavior of PutHKT2;1 from *P. tenuiflora* and TsHKT1;2 from *Thellungiella salsuginea* [40, 46]. The K^+^/Na^+^ transport properties of transgenic *Arabidopsis* were also confirmed in *MeHKT1* transgenic yeast (Fig. 4). Similar outcomes were previously documented in yeast expressing *RtHKT1* from *Reaumuria trigyna* [33]. These findings underscore the remarkable influence of external K^+^ and Na^+^ on the ion transport HKTs properties.

The reduction of Na^+^ accumulation in plant shoots and the maintenance of a balanced homeostasis between K^+^ and Na^+^ in plant cells are of paramount importance for ensuring the survival and growth of plants under salt-stress conditions [50]. HKT1-type transporters play a pivotal role in facilitating plant adaptation to salt stress. Research indicates that HKT1-type transporters are involved in regulating the distribution of Na^+^ within plants. They achieve this by actively transporting Na^+^ from the root xylem, thereby enhancing plant salt tolerance through the reduction of Na^+^ levels in the shoots [50, 51]. Previous research has demonstrated that OsHKT1;5 in rice and TmHKT1;5-A and TaHKT1;5-D in wheat could all increase salt tolerance by excreting Na^+^ from the root xylem and lowering Na^+^ accumulation in shoots [27, 31, 37]. Interestingly, vascular-specific expression of *AtHKT1;1* improves salt tolerance by decreasing Na^+^ accumulation in shoots [21, 23, 47, 52]. In contrast, *Arabidopsis* is vulnerable to salt stress due to constitutive expression of *AtHKT1*;*1* [53]. In the present study, *MeHKT1* transgenic *Arabidopsis* accumulates more Na^+^ in roots and shoots compared to WT under salt stress. On the other hand, their K^+^ contents remain unchanged, resulting in a lower K^+^/Na^+^ ratio and weaker growth of transgenic plants than WT (Figs. 6 and 8). Moreover, chlorophyll, proline and malondialdehyde (MDA) contents in salt-stressed plants are commonly used to characterize plant salt tolerance. The chlorophyll and proline contents of the transgenic plants were substantially lesser than in WT. This suggests that the transgenic plants exhibit decreased photosynthesis but also experience more osmotic stress compared to the WT. However, MDA content in transgenic plants was notably increased in comparison to WT, indicating that the disruption of membrane lipids is more noticeable in the transgenic plants in comparison to WT (Fig. 8). These findings indicate that MeHKT1 may transport Na^+^ into plant cells and negatively regulate salt tolerance under salt stress. These results align with prior research where HvHKT1;5 was discovered to be engaged in the loading of Na^+^ from roots into shoots via the xylem, ultimately leading to a negative regulation of salt tolerance of barley [34].

## Conclusions

In summary, this study demonstrates that cassava high-affinity potassium transporter 1 (*MeHKT1*) exhibits similar expression patterns in roots, stems, and leaves, with its transcription being induced by salt stress, particularly in roots where it has the highest transcription. Moreover, cassava MeHKT1 acts as a membrane protein for K^+^ uptake in yeast and *Arabidopsis* under low potassium (K^+^) conditions. However, MeHKT1 acts as a negative regulator of salt stress in transgenic *Arabidopsis* plants, impeding plant growth in response to salt stress and exhibiting a reduced K^+^/Na^+^ ratio relative to wide type (WT) plants. Therefore, this study investigated the roles of MeHKT1 in response to low potassium and high salt stress in detail and *MeHKT1* holds potential as a candidate gene for genetic editing to breed salt-tolerant crop varieties.

## Methods

### Plant materials and growth conditions

Cassava (*Manihot esculenta* Crantz SC8) histocultured seedlings were placed in 1/2 MS solid medium. Subsequently, they were cultured within a plant light incubator for 50 d, maintaining controlled environmental conditions throughout. These conditions encompassed an incubation temperature of 25 ± 2℃, a photoperiod of 14 h of light subsequent with 10 h of darkness, relative humidity of 65 ± 5%, and optical density of 150 µmol·m^−2^·s^−1^. Healthy cassava seedlings of the same sizes were selected and immersed in an Afdaling nutrient solution [54] for 1 week for acclimatization. Subsequently, they were transferred to a medium containing 50 µM KCl or 150 mM NaCl for 48 h to induce stress. Four replicates were established for each treatment, and samples were assembled during distinct time periods: 0, 3, 6, 12, 24, and 48 h, respectively. These samples were divided into roots, stems, and leaves for collection. They were rapidly frozen using liquid nitrogen and preserved at -80 °C to facilitate subsequent RNA extraction.

*Arabidopsis thaliana* (*Arabidopsis thaliana* (L.) Heynh) ecotype Columbia (Col-0) plants were used as the WT, and its seeds, which are kept in our laboratory, were sterilized with 10% sodium hypochlorite for 10 min and rinsed 5 times in sterile ddH_2_O. Afterward, the seeds were vernalized at a temperature of 4 °C for 3 d in the dark. The vernalized seeds were then added to a 1/2 MS solid medium, vertically orientated. They were then positioned in a plant light incubator at 22 °C, with a photoperiod of 16 h of light followed by 8 h of darkness. The relative humidity was maintained at 70%. After 5 d in these conditions, the seedlings were transplanted into pots filled with nutrient soil, specifically a 1:1 mixture of vermiculite and peat. They continued to grow under the same environmental conditions as mentioned above.

### Cloning and bioinformatics analysis of the MeHKT1

Extraction of total RNA from cassava seedlings was performed using the methodology specified in the RNAprep Pure Plant kit (TIANGEN, Beijing, China). The quality of RNA was assessed utilizing the Agilent 2100 Bioanalyzer and agarose gel electrophoresis. For further analysis, first-strand cDNA was synthesized utilizing the PrimeScript II 1st Strand cDNA Synthesis Kit (TaKaRa, Japan). This cDNA served as a template for the subsequent amplification of the target gene. The PCR product was cloned into the T vector according to the instructions manual of the pEASY®-Blunt Cloning Kit (Trans Gen, Beijing, China) and then sequenced by Shanghai Bioengineering Co. for verification. The obtained *MeHKT1* sequence was used to determine the open reading frame and the putative amino acid sequence using the ORFfinder available at the NCBI website (http://www.ncbi.nlm.nih.gov/gorf/gorf.html).

Multiple alignments of amino acid sequences were performed using DNAMAN 6.0 software. The Predictions of transmembrane structural domains of the MeHKT1 protein were obtained using the online software DeepTMHMM (https://dtu.biolib.com/app/DeepTMHMM/run). The neighbor-joining method with 1,000 bootstrap replicates was employed for phylogenetic analysis using the MEGA11.0 software.

### RT-PCR and qRT-PCR analysis

Total RNA was isolated from each sample including roots, stems, and leaves treating at different time points utilizing an RNA extraction kit (TIANGEN, Beijing, China), adhering to the guidelines provided by the manufacturer. First-strand cDNA was synthesized from 2 µg of RNA utilizing the PrimeScript II 1st Strand cDNA Synthesis Kit (TaKaRa, Japan). To investigate the spatial expression of *MeHKT1*, cDNA served as a template for PCR amplification. The amplification was carried out using MeHKT1-specific primers, MeHKT1-semiF and MeHKT1-semiR (refer to Supplementary Table S1), with *MeActin* as an internal control. The following reaction conditions were employed: initial denaturation at 95 °C for 5 min, 28 cycles consisting of denaturation at 94 °C for 15 s, annealing at 56 °C for 15 s, and extension at 72 °C for 10 s. This was concluded with a final extension at 72 °C for 10 min. To further investigate the transcript level of the *MeHKT1* gene, qRT-PCR was executed and carried out utilizing the Real Universal Color PreMix SYBR Green (TIANGEN, Beijing, China), along with MeHKT1-specific primers (MeHKT1-qRT-F and MeHKT1-qRT-R, as detailed in Supplementary Table S1). *MeActin* was employed as an internal reference gene. The qRT-PCR reactions were performed on a Qiagen Rotor-gene Q real-time PCR instrument (Qiagen, Germany) under the following conditions: an initial denaturation at 95 °C for 15 min, followed by 40 cycles involving denaturation at 95 °C for 10 s and annealing/extension at 60 °C for 30 s. The relative expression level of the *MeHKT1* gene was calculated by the comparative 2^−△△CT^ method. All experiments encompassed three technical replicates and four biological replicates.

### Subcellular localization analysis

To explore the subcellular localization of MeHKT1, the *MeHKT1* ORF, eliminating the stop codon, was amplified utilizing MeHKT1-specific primers (MeHKT1-GFP-F and MeHKT1-GFP-R, as detailed in Supplementary Table S1). Subsequently, the amplified product and the vector pCAMBIA1300-35 S-GFP were digested with *BamH* I and *Sma* I, respectively. The resulting target fragments were then recovered and ligated to construct the recombinant vector pCAMBIA1300-35 S-MeHKT1-GFP. The recombinant plasmid and the pCAMBIA1300-35 S-GFP plasmid were then transformed into *Agrobacterium tumefaciens* strain GV3101. GV3101 carrying either the fusion vector (35 S::MeHKT1-GFP) or pCAMBIA1300-35 S-GFP (35 S::GFP) was utilized to transiently transform tobacco (*Nicotiana benthamiana*) leaves. After incubation for 3 days, GFP fluorescence was observed using a fluorescence confocal microscope (FV3000; Olympus Corporation, Tokyo, Japan) as previously reported [55].

### Examination of MeHKT1 function in yeast

The full-length coding sequence of *MeHKT1* was amplified with p416-MeHKT1-F and p416-MeHKT1-R primers (Supplementary Table S1). This sequence was then inserted into the p416 vector containing the GPD promoter to investigate the function of MeHKT1 in yeast. The recombinant p416-MeHKT1 plasmid was introduced into the yeast mutant strains CY162 (*trk1Δtrk2Δ*) by LiAc/PEG mediated method, following the procedure outlined in a previous report [56]. The yeast strain CY162, which bears mutations in K^+^ ion transporters TRK1 and TRK2, exhibited sensitivity to K^+^ deficiency. The transgenic yeast cells were cultured in liquid YPD medium at 28℃ and 200 rpm until OD_600_ = 1.0 ~ 1.5 for the yeast growth assay. Then, 100 µL of yeast culture medium was diluted to 10-fold, 100-fold, and 1000-fold, respectively. Subsequently, 5 µL of the culture solution was dispensed on AP (8 mM phosphoric acid, 10 mM arginine, 2% glucose, 2 mM MgSO_4_, 1 mM KCl, 0.2 mM CaCl_2_, plus trace elements and vitamins, pH = 6.5) solid medium with different K^+^ concentration or 50 mM NaCl concentration and cultured in inverted mode at 28℃ for 3–5 d. Simultaneously, the growth phenotypes of yeast cells transformed with the empty vector p416-GPD served as a control for comparison.

### Functional analysis of transgenic Arabidopsis thaliana

The full length of *MeHKT1* gene was amplified with 1300-MeHKT1-F and 1300-MeHKT1-R primers (Supplementary Table S1). This sequence was then inserted into the pCAMBIA1300 vector carrying the hygromycin B (HygB) resistance gene. pCAMBIA1300-MeHKT1 recombinant vector were transformed into *Agrobacterium tumefaciens* strain GV3101. Subsequently, wide type (WT) *Arabidopsis thaliana* was transformed by applying the *Agrobacterium*-mediated technique as previously described [57]. Transgenic *Arabidopsis* plants were initially screened by exposing them to a Hygromycin B (HygB) concentration of 50 mg/L. Further confirmation was carried out through PCR amplification. The transgenic *Arabidopsis* plants overexpressing *MeHKT1* were labeled “OE” and homozygous transgenic lines of the T3 generation were utilized for subsequent investigations.

To assess the impact of low K^+^ or salt stress on the growth of WT and transgenic *Arabidopsis*, 4-day-old seedlings were transferred to 1/2 MS solid medium containing 50 µM KCl or 75 mM NaCl. The low K^+^ medium was determined by modifying the normal MS medium (NH_4_NO_3_ instead of KNO_3_ and NH_4_H_2_PO_4_ instead of KH_2_PO_4_). The final K^+^ concentration was adjusted by adding KCl. Subsequently, these seedlings were placed vertically in an incubator at 22 °C to continue the growth for two weeks before photographing. Finally, measurements of the length of the primary root and fresh weight were taken, and ion content was determined.

To further verify the salt tolerance of transgenic *Arabidopsis thaliana* in soil, transgenic *Arabidopsis thaliana* and WT *Arabidopsis thaliana* seedlings grown vertically on 1/2 MS medium for 5 d were transplanted to soil and placed in a greenhouse at 22 °C under a 12 h light/12 h dark cycle. These seedlings were allowed to grow for 8 d before undergoing salt treatment. The salt treatment involved watering with 2 L of a 300 mM NaCl solution in the treatment group, while an equal volume of watering was utilized for the control group. After 3 weeks of treatment with salt, the seedlings were photographed, and relevant parameters were determined.

### Assessment of related physiological indexes

The quantification of malondialdehyde (MDA) and proline contents was conducted in accordance with the protocols presented in the MDA and proline assay kits provided by Nanjing Jiancheng Bioengineering Institute. 0.1 g of WT and transgenic plant leaves were placed in centrifuge tubes (5 mL) to determine chlorophyll content. The leaves were completely ground with steel balls and extracted using 3 mL of 80% acetone. Following a 3-h incubation at room temperature in the absence of light, the mixture was subjected to centrifugation at 12,000 rpm for 10 min to collect the supernatant from the extract. The absorbance values of the supernatant extract were calculated at 663 nm and 645 nm utilizing a UV spectrophotometer, with the absorbance value of 80% acetone as a blank control. Chlorophyll concentration was calculated following the already established procedure [58].

### Ion content determination

The collected plant samples were initially rinsed with distilled water to determine the ion content and dried in an oven at 80 °C until constant weight. These dried samples were then subjected to digestion in 10% HCl. Subsequently, the concentrations of K^+^ and Na^+^ in the digested solution were determined utilizing a flame atomic absorption spectrophotometer as previously reported [59].

### Statistical analysis

All experiments were carried out with a minimum of three independent biological replicates. Statistical analysis was carried out utilizing SPSS 20 software, and statistical differences were assessed utilizing one-way analysis of variance (ANOVA). *P* < 0.05 reflected a significant difference. The outcomes were presented as mean ± SD.

### Supplementary Information

Supplementary Material 1.

Supplementary Material 2.

Supplementary Material 3.

## Data Availability

The datasets used and/or analyzed during the current study are available from corresponding authors on reasonable request.
