# Supplementary material for: A high-affinity potassium transporter (MeHKT1) from cassava (Manihot esculenta) negatively regulates the response of transgenic Arabidopsis to salt stress
Source: BMC Plant Biol. 2024 May 7;24:372. doi: 10.1186/s12870-024-05084-7 (PMC11075273; doi:10.1186/s12870-024-05084-7)
Supplement: Supplementary file 1 — Supplementary Material 1. [file 12870_2024_5084_MOESM1_ESM.docx]

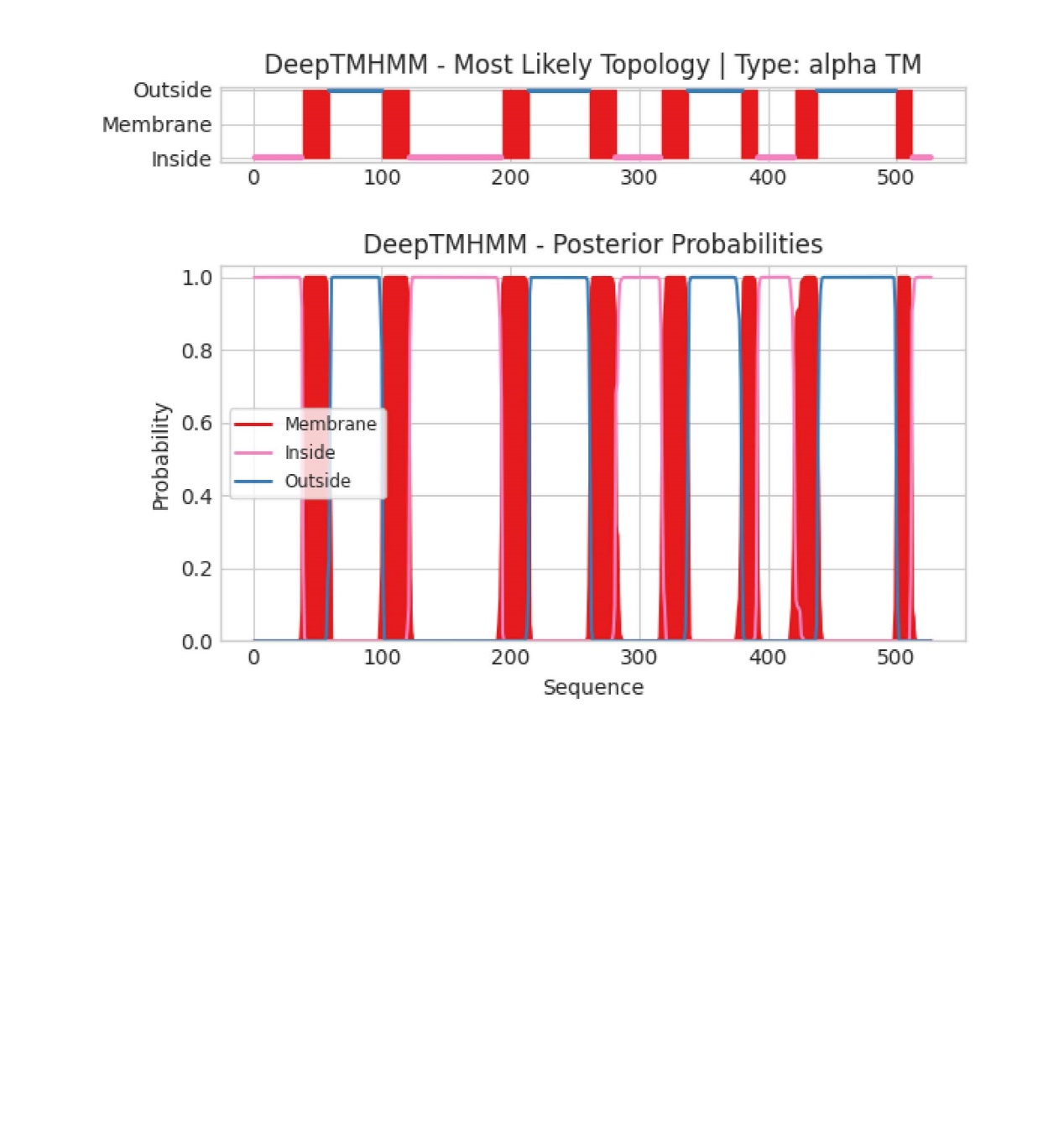


Figure S1. Prediction of the MeHKT1 transmembrane domain.

The putative transmembrane domain of MeHKT1 was obtained by means of the online software DeepTMHMM (https://dtu.biolib.com/app/DeepTMHMM/run).
