# Supplementary material for: A high-affinity potassium transporter (MeHKT1) from cassava (Manihot esculenta) negatively regulates the response of transgenic Arabidopsis to salt stress
Source: BMC Plant Biol. 2024 May 7;24:372. doi: 10.1186/s12870-024-05084-7 (PMC11075273; doi:10.1186/s12870-024-05084-7)
Supplement: Supplementary file 2 — Supplementary Material 2. [file 12870_2024_5084_MOESM2_ESM.docx]

MeHKT1


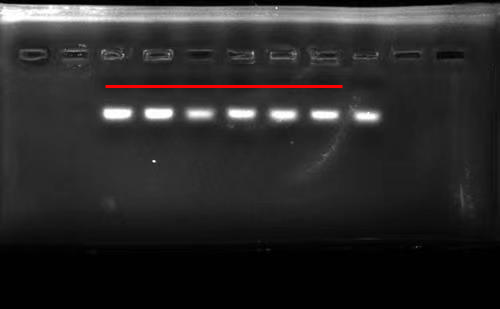


MeActin


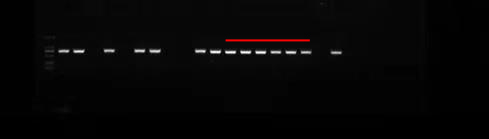


Figure 2.The expression patterns of the *MeHKT1* gene in cassava. (A) RT-PCR analysis of *MeHKT1* gene in various tissues of cassava. *Actin* was used as an internal control.
