# Supplementary material for: A high-affinity potassium transporter (MeHKT1) from cassava (Manihot esculenta) negatively regulates the response of transgenic Arabidopsis to salt stress
Source: BMC Plant Biol. 2024 May 7;24:372. doi: 10.1186/s12870-024-05084-7 (PMC11075273; doi:10.1186/s12870-024-05084-7)
Supplement: Supplementary file 3 — Supplementary Material 3. [file 12870_2024_5084_MOESM3_ESM.docx]

| **Table S1. Primer sequences used in the experiment.** | |
| --- | --- |
| *MeActin*-F | TGTTCCCTGGCATTGCAGAC |
| *MeActin*-R | CTCGTCGTACTCGCCCTTG |
| *MeHKT1*-semiF | CTTGGAGGATTAGTTTCGGC |
| *MeHKT1*-semiR | GGCCTGAGTTCTTGCTAAAG |
| *MeHKT1*-qRT-F | TCTACAGGTTCATCCTTGTCC |
| *MeHKT1*-qRT-R | CTGTTGTTGCAGACACAGATG |
| p416-MeHKT1-F | CGGGATCCGATGAAGGACTCGTTCCCTTG |
| p416-MeHKT1-R | TCCCCCGGGTTACAGAAGTATCCAAGCTCTACC |
| *MeHKT1*-GFP-F | AGGACTAGTATGAAGGACTCGTTCCCTTG |
| *MeHKT1*-GFP-R | CGGGATCCCAGAAGTATCCAAGCTCTACCTC |
| 1300-MeHKT1-F | AGGACTAGTGATGAAGGACTCGTTCCCTTG |
| 1300-MeHKT1-R | CGGGATCCTTACAGAAGTATCCAAGCTCTACC |
